# Catalyst‐Free Transformation of Carbon Dioxide to Small Organic Compounds in Water Microdroplets Nebulized by Different Gases

**DOI:** 10.1002/advs.202406785

**Published:** 2024-08-11

**Authors:** Masoud A. Mehrgardi, Mohammad Mofidfar, Jia Li, Christian F. Chamberlayne, Stephen R. Lynch, Richard N. Zare

**Affiliations:** ^1^ Department of Chemistry Stanford University Stanford California 94305 USA; ^2^ Department of Chemistry University of Isfahan Isfahan 81746 Iran; ^3^ College of Chemical Engineering Shijiazhuang University Shijiazhuang 050037 China

**Keywords:** aerosols, carbon dioxide, carbon dioxide reduction, formic acid, water, water microdroplets, water‐suppressed NMR

## Abstract

A straightforward nebulized spray system is designed to explore the hydrogenation of carbon dioxide (CO_2_) within water microdroplets surrounded by different gases such as carbon dioxide, nitrogen, oxygen, and compressed air. The collected droplets are analyzed using water‐suppressed nuclear magnetic resonance (NMR). Formate anion (HCOO^−^), acetate anion (CH_3_COO^−^), ethylene glycol (HOCH_2_CH_2_OH), and methane (CH_4_) are detected when water is nebulized. This pattern persisted when the water is saturated with CO_2_, indicating that CO_2_ in the nebulizing gas triggers the formation of these small organics. In a pure CO_2_ atmosphere, the formate anion concentration is determined to be ≈70 µm, referenced to dimethyl sulfoxide, which has been introduced as an internal standard in the collected water droplets. This study highlights the power of water microdroplets to initiate unexpected chemistry for the transformation of CO_2_ to small organic compounds.

## Graphical abstract

Water microdroplets surrounded by carbon dioxide can transform this greenhouse gas into small organic compounds such as formic acid, acetic acid, methane, and ethylene glycol, which addresses the challenge of global warming.

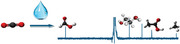

## 1 Introduction

The transformation of carbon dioxide to small organic compounds has been strongly motivated by two factors, which are related to clean energy production. One of them is the possibility of the capture of carbon dioxide so that it is turned into useful small organic molecules that can be used as feedstocks and fuels.^[^ ^1^ ^]^ As the climate change crisis intensifies and carbon dioxide levels in the atmosphere exceed 400 ppm, CO_2_ capture strategies take center stage in our battle against global warming. Moreover, the approaches that involve the hydrogenation of CO_2_ to create hydrogen storage systems, such as formic acid with a volumetric hydrogen density of 53 g of H_2_ per liter, represent a promising method for storing hydrogen, a sustainable and renewable energy source.^[^ ^1^, ^2^ ^]^

Presently, organic molecules are formed from carbon dioxide in many ways that involve the use of electrochemical,^[^ ^3^ ^]^ photoelectrochemical (PEC),^[^ ^4^, ^5^ ^]^ photocatalysis,^[^ ^6^ ^]^ electrocatalysis,^[^ ^7^ ^]^ and biochemical^[^ ^8^ ^]^ techniques. Moreover, the transformation of carbon dioxide to small organic compounds in charged microdroplets, in electrospray, and in nanoESI has been explored recently.^[^ ^9^, ^10^, ^11^ ^]^ In sharp contrast, the work presented here describes the production of organic molecules from dissolved carbon dioxide in water without the use of any catalyst or external electrochemical energy source. This task is accomplished by aqueous microdroplet chemistry, which only requires the energy to generate a spray.

It is becoming increasingly well established that reactants in microdroplets produce different chemistry than the same reactants in bulk solution. One of the first observed phenomena was that many reactions are accelerated in microdroplets compared with bulk solution.^[^ ^12^, ^13^ ^]^ Additionally, unusual reductions have been observed in microdroplets without the presence of a known reducing agent, such as nanoparticles from gold (III) chloride salts,^[^ ^14^ ^]^ the reductions of a number of organic acids, such as pyruvate to lactate,^[^ ^15^ ^]^ and the catalyst‐free production of hydrogen peroxide (an oxidizing agent) in water microdroplets.^[^ ^16^, ^17^ ^]^ In this study we show that water microdroplets in contact with carbon dioxide (CO_2_) produce some small organic species including, formate and acetate ions, ethylene glycol, and even methane. The identification of these compounds is verified by water‐suppressed nuclear magnetic resonance (NMR) studies of the collected droplets. This process is achieved using sprayed water microdroplet chemistry in contact with different nebulizing gases.

## 2 Results and Discussion

**Figure 1:**
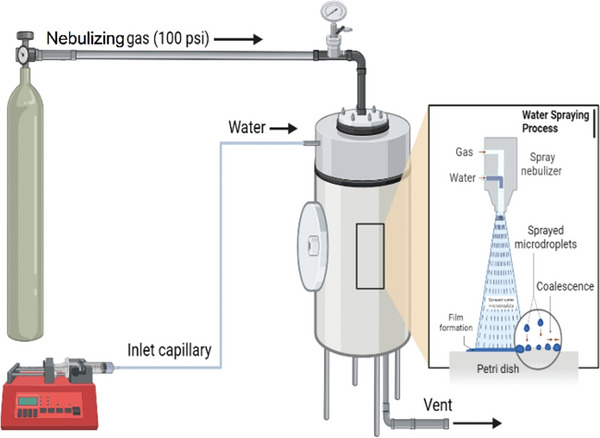
Microdroplet generation and collection apparatus.

**Figure** **1** presents an experimental schematic of a simple nebulized spray setup for catalyst‐free hydrogenation of CO_2_ in water microdroplets. To avoid contamination from volatile organic species that are present in laboratory air, we constructed a closed stainless‐steel chamber for the microdroplet sprays. The chamber was cycled between vacuum and nebulizing gas three times prior to running the experiment. Microdroplets were generated from water exiting a glass capillary into rapidly flowing different sheath gases. As a further safeguard, we relocated the entire experiment to a room that was not being used for any other experimentation.

The control samples were recorded under nearly identical conditions, with the sheath gas turned off so the water dripped out of the capillary in large droplets instead of as a microdroplet spray. Each sample was paired with a control that was taken using the same syringe of water as the sprayed sample. The experiment was carefully designed such that the control sample touched all the same surfaces as the sprayed sample.

**Figure 2:**
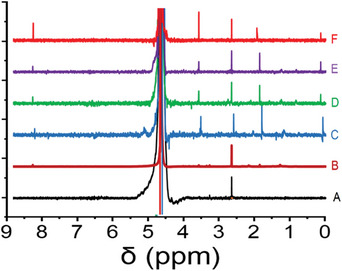
Water‐suppressed NMR of unsprayed control under nitrogen atmosphere A), removed CO_2_ water by purging nitrogen and sprayed by nitrogen B), water microdroplets sprayed by nitrogen C), oxygen D), compressed air E) and carbon dioxide F) sheath gases. Samples are normalized to the DMSO peak area.

**Figure 3:**
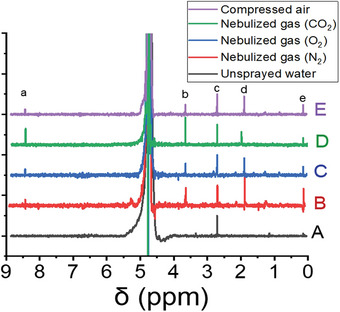
Water‐suppressed NMR of water microdroplets sprayed by nitrogen A) and subsequently spiked by 200 µm formic acid B), 40 µm ethylene glycol C), 40 µm acetic acid D), and 1 s methane purge E). Samples are normalized to the DMSO peak area. Labels (a–e) are formic acid, ethylene glycol, DMSO (internal standard), acetic acid, and methane, respectively.

Water‐suppressed NMR allows for an NMR spectrum to be taken without deuterated water as the solvent. In water‐suppressed NMR, a radio frequency pulse at the adsorption frequency for water is sent just prior to a typical pump pulse for NMR. This pre‐saturation pulse saturates the water transition, thereby suppressing its intensity in the NMR spectrum.^[^ ^18^ ^]^ A small amount of deuterated water is still needed to lock the NMR. Deuterated water was added to the sample in the NMR tubes to bring the final sample to a 9:1 H_2_O to D_2_O water mixture. All concentrations reported in this paper are concentrations in the collected sample and have been corrected for this dilution. **Figure** **2** shows water‐suppressed NMR spectra of collected microdroplets nebulized with nitrogen, oxygen, carbon dioxide, compressed air, and unsprayed water as well. Several different peaks are observed at the chemical shifts of 8.40, 3.66, 2.02, and 0.13 ppm which can be attributed to the formation of formate, ethylene glycol, acetate, and methane, respectively. All assignments were confirmed by spiking a sample with formic acid, ethylene glycol, and acetic acid and sparging with methane (**Figure** **3**). We found that the respective peaks increased in magnitude but did not split. Regarding Figure 3, it is worth noting that the peaks for acetic acid and formic acid shift with the pH of the solution. **Table** **1** lists the calculated concentrations for different generated compounds in microdroplets nebulized by means of different gases. As the results demonstrate, the concentration of formic acid and acetic acid dramatically increases when carbon dioxide is the nebulizing gas. The formation of these compounds, even when CO_2_ is absent in the nebulizing gas, highlights the role of pre‐dissolved carbon dioxide in this process. Prior to spraying, the water had been exposed to the atmosphere and had an equilibrium amount of dissolved carbon dioxide in it. Treating the atmosphere as an infinite source of CO_2_ at 410 ppm, we can use Henry's constant (0.034 mol kg bar^−1^ for CO_2_ in water)^[^ ^19^ ^]^ to obtain the amount of dissolved CO_2_ gas. It is calculated to be 13.9 µm. There is also a pH‐dependent equilibrium with carbonic acid and its derivatives similarly present in the micromolar range, which is discussed excellently elsewhere.^[^ ^19^ ^]^

**Table 1:** Concentrations of different generated compounds in microdroplets are nebulized by means of different gases.

Gas	[HCOO^−^]	[HOCH_2_CH_2_OH]	[CH_3_COO^−^]	[CH_4_]
CO_2_	71.1	19.2	48.5	4.6
N_2_	22.1	19.5	12.2	13.7
O_2_	14.2	8.8	12.5	6.0
Air	14.6	6.5	17.1	8.2

In a control experiment, we reduced the concentration of pre‐dissolved carbon dioxide by sparging nitrogen while boiling the water. As illustrated in Figures 2 and 3, the formation of all compounds decreased with the reduction of dissolved carbon dioxide. This observation provides strong evidence for the role of dissolved carbon dioxide in the formation of various organic compounds. On the other side, the promotion of CO_2_ transformation when carbon dioxide is used as a nebulizing gas confirms the important role of gas phase CO_2_.

**Figure 4:**
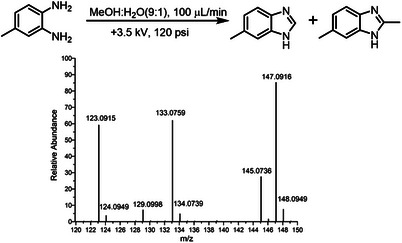
Tandem mass spectrum with energy of 30 eV by ion trap mode.

The transformation of CO_2_ to formic and acetic acid was further investigated using mass spectrometry. 3,4‐diaminotoluene (0.011 mg, 90 µm) dissolved in 500 µL water and methanol (v:v, 1:9) act as traps for formic and acetic acid respectively via direct injection mass spectrometry (**Figure** **4**). The peaks at *m/z* 133.0759 (δ = 0.75 ppm) and 147.0916 (δ = 0.68 ppm) are products of 6‐methylbenzimidazole and 2,6‐dimethylbenzimidazole, respectively. The reagent of 3,4‐diaminotoluene resides in the reaction system from the mass spectrum. To confirm the two products, collision‐induced dissociation tandem mass spectrometry is used with an energy of 30 eV by ion trap mode. Both the fragments resulting from the loss of methyl are found at peaks *m/z* 118.09 and 132.00.

Having established the presence of small organic compounds in sprayed water droplets containing dissolved CO_2_, we speculate how it might be formed. It is well known that CO_2_ can be converted electrochemically to these compounds.^[^ ^20^, ^21^, ^22^, ^23^, ^24^, ^25^, ^26^, ^27^ ^]^ We propose that when CO_2_ is dissolved in water, the bicarbonate anion (HCO_3_^−^) is preferentially adsorbed at the surface of the water microdroplet (Figure S1, Supporting Information). This behavior is consistent with the observation that microbubbles are negatively charged.^[^ ^28^, ^29^ ^]^ We also sprayed D_2_O instead of H_2_O and detected deuterated formic, bicarbonate, and acetic acid to provide strong evidence that formate, bicarbonate, and acetate anions are not coming from impurities (Figure S2, Supporting Information). The negative surface charge gives rise to an electric double layer inside the droplet.^[^ ^30^ ^]^ In turn, the electric double layer can drive electrochemical reactions, as recent calculations have suggested.^[^ ^31^, ^32^ ^]^ We propose that the electric field drives the transfer of an electron from bicarbonate anion to dissolved CO_2_ thereby transferring an electron from the surface to the interior volume. This transfer generates the carbon dioxide radical anion (·CO_2_^−^), which, while transient, is also a major player in electrochemical reductions of CO_2_.^[^ ^23^ ^]^ It is possible ·CO_2_^−^ further decomposes, replenishing the carbon dioxide and generating a hydroxyl radical.

Once the CO_2_ radical anion is present (or, alternately, the hydroxyl radical) there are several radical chain mechanisms that can lead to the formation of formate anion. As shown in **Table** **2**, starting from the CO_2_ radical anion, a hydrogen is extracted from water to form the formate anion and hydroxyl radical. The hydroxyl radical then extracts an alcohol group from bicarbonate anion to regenerate the initial CO_2_ radical anion as well as hydrogen peroxide. We also propose an alternate radical chain mechanism. Starting from the protonated CO_2_ radical, a hydride shift of the hydrogen from the oxygen to the carbon generates the formyl radical. The formyl radical extracts a hydrogen atom from water to make the hydroxy radical. The hydroxy radical can extract an alcohol group from carbonic acid to regenerate the initial protonated CO_2_ radical as well as hydrogen peroxide. Given the pH gradients found in microdroplets, it is entirely possible that both mechanisms are present at different locations in the microdroplet.^[^ ^30^ ^]^ Furthermore, given the results that underscore the significant role of gas phase CO_2_, we suggest an additional pathway where gas phase CO_2_ can interact with ·OH at the water‐air interface of microdroplets. We also propose the formation of different radicals for further hydrogenation of CO_2_ to other compounds (Table 2). The reaction: OH^−^ + H_3_O^+^ →·OH + H· + H_2_O (Table 2, Pathway 1), is the only one that produces the key reducing H· atom. Because the reaction: OH^−^ + H_3_O^+^→ ·OH + H· + H_2_O, is thermodynamically forbidden in bulk water, it must happen at the surface of microdroplets.^[^ ^33^ ^]^ Clearly more experiments are needed to determine the mechanism for formic acid formation in water droplets, but the present results serve as another remarkable example of the power of water microdroplets to promote redox reactions.

**Table 2:** Proposed possible mechanisms for hydrogenation of CO_2_.

Proposed mechanisms for formic acid formation
Pathway 1: Bicarbonate anion pathway
OH⁻ + H_3_O^+^ → ·OH + H· + H_2_O CO_2_ + H_2_O → H_2_CO_3_ → HCO_3_⁻ + H_3_O^+^ HCO_3_⁻ + ·OH → ·COO⁻ + H_2_O_2_ ·COO⁻ + H_2_O→ HCOO⁻ + ·OH

Pathway 2: Carbonic acid pathway
H_2_CO_3_ + ·OH → ·COOH + H_2_O_2_ ·COOH + H_2_O→ HCOOH + ·OH HCOOH + H_2_O → HCOO⁻ + H_3_O^+^

Pathway 3: Gas phase CO_2_ pathway
CO_2_ + H· → ·COOH ·COOH+ H· → HCOOH HCOOH + H_2_O → HCOO⁻ + H_3_O^+^

Proposed mechanisms for ethylene glycol formation
Pathway 1
·COOH + ·COOH → HOOCCOOH HOOCCOOH + 2·H → HOCH(·O) CH(O·)OH HOCH(·O) CH(O·)OH+ 2·H→(HO)_2_CHCH(OH)_2_ (HO)_2_CHCH(OH)_2_ → O ═ CHCH ═ O + 2 H_2_O O ═ CHCH ═ O + 2·H→ ·OCHCHO· ·OCHCHO· + 2·H→ HOCH_2_CH_2_OH

Pathway 2
HCOOH+ H· → ·HCO+ H_2_O ·HCO+ H· → H_2_CO H_2_CO+ H· → ·CH_2_OH ·CH_2_OH + ·CH_2_OH → HO CH_2_CH_2_OH

Proposed mechanism for acetic acid formation
HOOCCOOH + H· → HOOCCH(‐O·)OH HOOCCH(‐O·)OH + H· →HOOCCH(OH)_2_ HOOCCH(OH)_2_→HOOCH(═ O) + H_2_O HOOCH(═ O) + H· → HOOCH_2_(O·) HOOCH_2_(O·) + ·OH →HOOCCH_2_· + H_2_O_2_ HOOCCH_2_· + H· → CH_3_COOH

Proposed mechanism for methane formation:
CH_3_COOH+ ·OH → CH_3_COO· + H_2_O CH_3_COO· → ·CH_3_ + CO_2_ ·CH_3_ + H· → CH_4_

## 3 Conclusion

In summary, we have observed the catalyst‐free hydrogenation of carbon dioxide in water microdroplets leading to the formation of different small organic molecules. These compounds were characterized by water‐suppressed NMR. This finding represents another striking example of how the chemistry in water droplets differs markedly from that of bulk water.

## 4 Experimental Section

### Materials

All water used was Corning Molecular Biology Grade Water (USP Sterile) water from Fisher Scientific. Dimethyl sulfoxide (DMSO) was also purchased from Fisher Scientific. Compressed nitrogen and carbon dioxide were provided by Praxair.

### Spray Setup and Chamber

The spray nozzle was constructed of a 1/16^th^ inch OD, 0.02‐inch ID stainless‐steel pipe with a concentric 360 µm OD, 100 µm ID glass capillary inside. The glass capillary poked out ≈1 mm past the end of the stainless‐steel piping. Water flows out the end of the capillary and 100 psi sheath gas of either nitrogen or carbon dioxide was delivered via the stainless‐steel piping around the capillary. The spray was directed downward toward a 35 mm diameter (10 mm high) polystyrene Petri dish. The distance from the glass capillary tip to the bottom of the Petri dish was 10 mm. For the Teflon collection, a 50 mm Teflon liner inside a 50 mm polystyrene Petri dish was used to capture the microdroplets. The spray distance of 10 mm was maintained; the same as the polystyrene collection. The spray nozzle was located inside a stainless‐steel chamber. The chamber was homebuilt from ultrahigh vacuum components. The chamber consisted mainly of a T joint of an 8‐inch diameter tube intersected with a 6‐inch diameter tube. The spray and collector were in the center of the 8‐inch tube and pointed down the axis of the tube. A door was located on the 6‐inch pipe end to access the collected samples after spraying. The chamber was vented while spraying to prevent pressure buildup from the sheathe gas.

### Experiment Workflow

The chamber was cycled between vacuum and carbon dioxide (alternately nitrogen in the nitrogen experiment) three times prior to each run. A syringe pump delivered 100 µL min^−1^ of water through the capillary. Sheathe gas of carbon dioxide (alternately nitrogen in the nitrogen experiment) was maintained at 100 psi. The spray was maintained for 15 min. After 15 min, the chamber was opened and 450 µL of the collected liquid was transferred to a quartz NMR tube. There was significant evaporation during this process, despite 1500 µL of water being sprayed only ≈50% was successfully collected.

A control sample was taken either immediately prior or immediately after each spray experiment (this was deliberately varied to prevent systemic bias). The same syringe full of water in the syringe pump was used for the control experiment as its paired sprayed experiment. The chamber was cycled between vacuum and carbon dioxide (alternately nitrogen in the nitrogen experiment) three times prior to each run. A syringe pump delivered 100 µL min^−1^ of water through the capillary. No sheath gas was supplied, so the water formed large droplets that fell off the end of the glass capillary into the Petri dish below. To avoid the large droplets forming on the glass capillary tip from contacting the stainless‐steel pipe, the glass capillary was extended to ≈5 mm past the end of the stainless‐steel tubing. This was run for 8 min to collect a similar volume of liquid as the sprayed samples; then the chamber was opened and 450 µL of the collected liquid was transferred to a quartz NMR tube.

To each 540 µL sample in the NMR tube, 60 µL of 90 µM DMSO in deuterated water was added and the NMR tubes and vortexed to ensure mixing. The dimethyl sulfoxide was used as an internal standard, and the deuterated water was necessary to lock the NMR signals water‐suppressed NMR spectra were acquired on a Varian Inova 600 MHz NMR spectrometer with a z‐gradient triple resonance HCN probe running VNMRJ 4.2 located in the Stanford Chemistry Department NMR facility. Pre‐saturation spectra were recorded with 32 scans over a spectral width of 9600 Hz at 25 °C with a recycle delay time of 1 s, then a saturation pulse of 5 s applied at the H_2_O frequency, followed by an ≈80° excitation pulse then an acquisition time of 4 s. NMR spectra were processed using MestReNova version 15.0.0‐34764. Spectra were rephased manually, baselined using the Whittaker Smoother algorithm, and normalized using the DMSO peak. Concentrations of formic acid were determined by the ratio of peak area between formic acid and the known DMSO concentration. Please note that formic acid and formate anion are in equilibrium and exchange so rapidly that only one NMR peak is observed. Acetic acid and acetate anion behave the same; this equilibrium causes the large pH–dependent shift in ppm that was experimentally observed.

## Conflict of Interest

The authors declare no conflict of interest.

## Author Contributions

The manuscript was written through the contributions of all authors. All authors have given approval to the final version of the manuscript.

## Supporting information

Supporting Information

## Data Availability

The data that support the findings of this study are available from the corresponding author upon reasonable request.
