# Supplementary material for: Catalyst‐Free Transformation of Carbon Dioxide to Small Organic Compounds in Water Microdroplets Nebulized by Different Gases
Source: Adv Sci (Weinh). 2024 Aug 11;11(38):2406785. doi: 10.1002/advs.202406785 (PMC11481208; doi:10.1002/advs.202406785)
Supplement: Supplementary file 1 — Supporting Information [file ADVS-11-2406785-s001.docx]

**Supplementary Material**

Catalyst-Free Transformation of Carbon Dioxide to Small Organic Compounds in Water Microdroplets Nebulized by Different Gases

Masoud A. Mehrgardi^a, b^, Mohammad Mofidfar^a^, Jia Li^c^, Christian F. Chamberlayne^a^, Stephen R. Lynch^a^, Richard N. Zare*^a^

^a^ Department of Chemistry, Stanford University, Stanford, California 94305 USA

^b^ Department of Chemistry, University of Isfahan, Isfahan 81746, Iran

^c^ College of Chemical Engineering, Shijiazhuang University, Shijiazhuang 050037, China

*Corresponding author: zare@stanford.edu

**Figures**


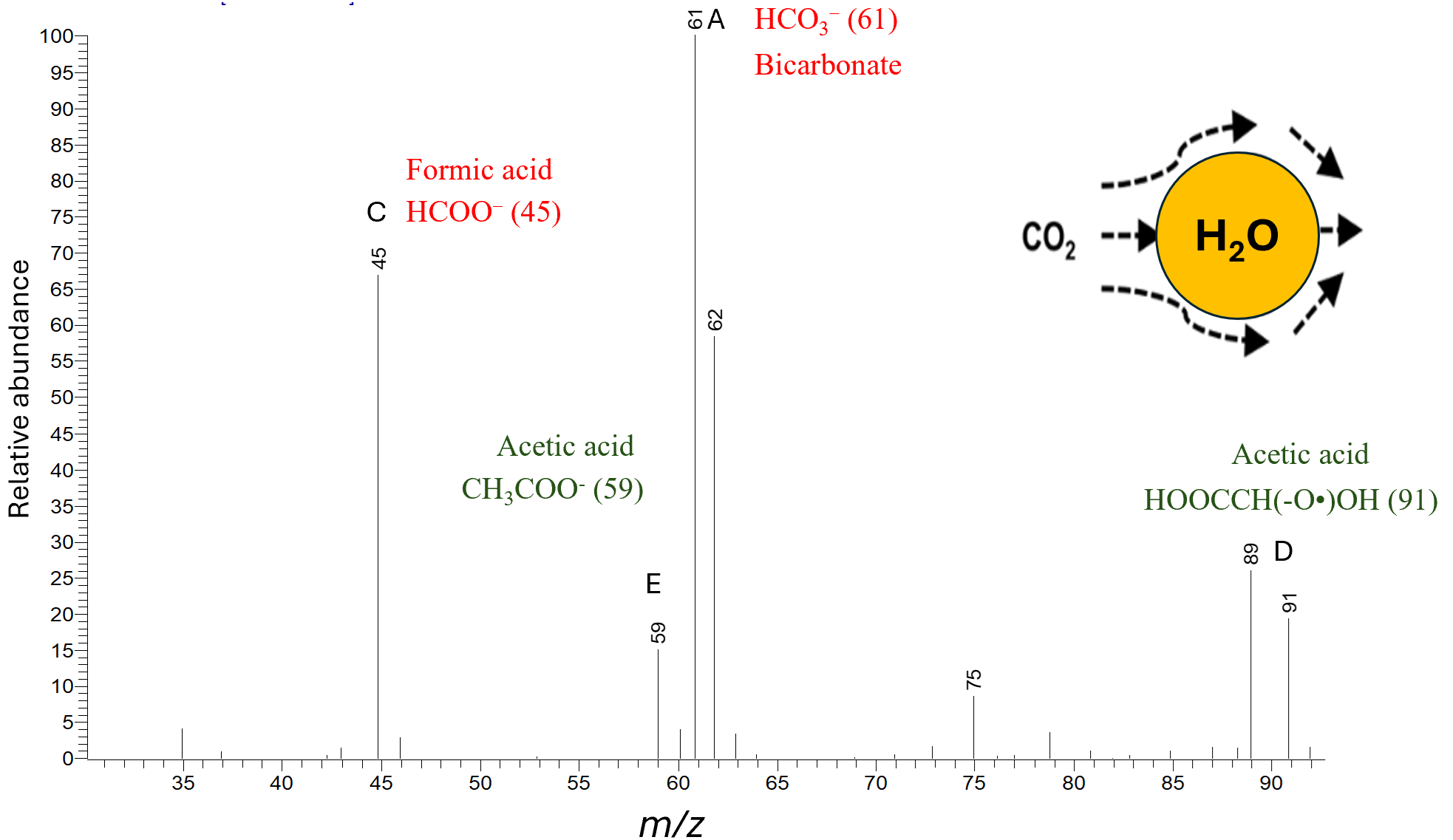


**Figure S1.** Evaluation of sprayed water microdroplets through direct detection of the formate and acetate anions in negative ion mode.


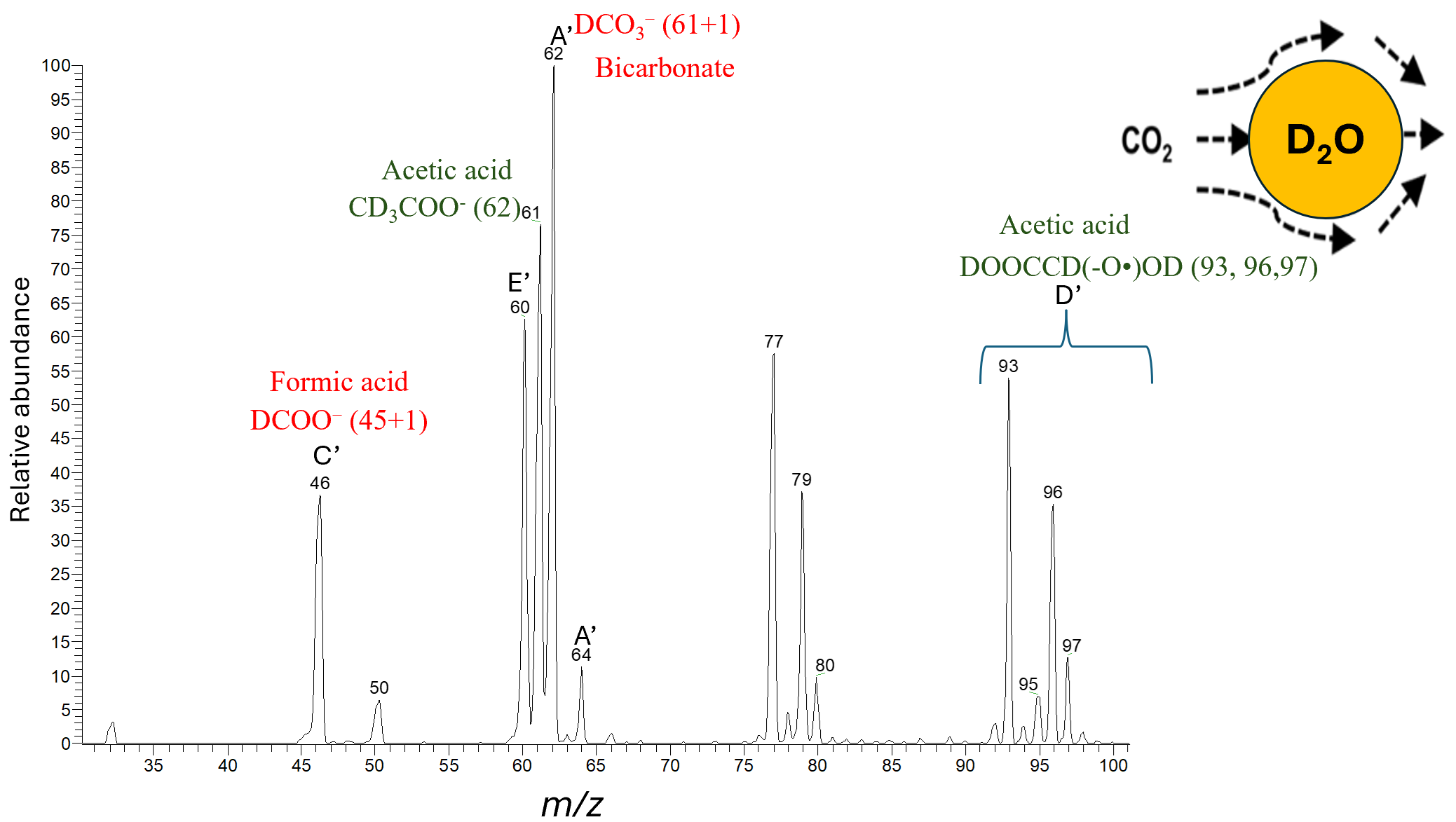


**Figure S2**. Evaluation of sprayed D2O microdroplets through direct detection of the formate and acetate anions in negative ion mode.
